# Serum Calcium and Magnesium Levels in Women Presenting with Pre-eclampsia: A Systematic Review and Meta-analysis Based on Observational Studies

**DOI:** 10.31661/gmj.v12i.3151

**Published:** 2023-12-18

**Authors:** Arqavan Eslamzadeh, Seyyed Mohammad amin Kashani, Nasrin Asadi, Sina Bazmi, Shahla Rezaei, Zeinab Karimimoghadam, Peyman Nowrouzi-Sohrabi, Reza Tabrizi

**Affiliations:** ^1^ Student Research Committee, Fasa University of Medical Sciences, Fasa, Iran; ^2^ Student Research Committee, Shiraz University of Medical Sciences, Shiraz, Iran; ^3^ Department of Obstetrics &Gynecology, School of Medicine, Shiraz University of Medical Sciences, Shiraz, Iran; ^4^ Nutrition Research Center, School of Nutrition and Food Sciences, Shiraz University of Medical Sciences, Shiraz, Iran; ^5^ Noncommunicable Diseases Research Center, Fasa University of Medical Sciences, Fasa, Iran; ^6^ Razi Herbal Medicines Research Center, Department of Biochemistry, School of Medicine, Lorestan University of Medical Sciences, Khorramabad, Iran; ^7^ USERN Office, Fasa University of Medical Sciences, Fasa, Iran; ^8^ Clinical Research Development Unit, Valiasr Hospital, Fasa University of Medical Sciences, Fasa, Iran

**Keywords:** Calcium, Female, Humans, Magnesium, Pre-eclampsia, Pregnancy

## Abstract

Background: Multiple studies have investigated the serum concentrations of calcium (Ca) and magnesium (Mg) in preeclampsia, but the results have been contradictory. The objective of this systematic review and meta-analysis was to examine the association between serum calcium and magnesium levels in patients with preeclampsia and those in the healthy pregnancies.Materials and Methods: A comprehensive search was conducted in various online databases, including PubMed/Medline, Scopus, Embase, Web of Sciences, and Cochrane library to identify relevant studies on Ca and Mg levels in preeclampsia up to July 2023. Inter-study heterogeneity across the included studies was assessed using the chi-square test and I2 statistic. Pooled effect size (ES) was calculated as weighted mean differences (WMDs) with corresponding 95% confidence intervals (CI).Results: A total of 76 articles (comprising 92 studies) were included, with a combined sample size of 10,482 participants (preeclampsia: n=3,991; controls: n=6,491). The random-effects model revealed significantly lower levels of calcium (WMD=-0.807 mg/dL, 95% CI: -0.983, -0.632, P0.01) and magnesium (WMD=-0.215, 95% CI: -0.338, -0.092, P0.01) in women with pre-eclampsia compared to the control group. However, the overall pooled WMD for calcium and magnesium levels did not significantly change when individual studies were excluded one by one.Conclusion: This meta-analysis demonstrates that the circulating levels of calcium and magnesium in patients with preeclampsia are significantly lower than those in the control group.

## Introduction

Preeclampsia is an abnormality in pregnancy characterized by an increase in blood pressure levels and change in blood trace elements levels. Preeclampsia is commonly defined by a systolic blood pressure≥140 mmHg or diastolic pressure≥90 mmHg.

Additionally, proteinuria may serve as a marker for preeclampsia when protein level in a sample of urine exceeds 30 mg/dL [1][2]. Preeclampsia can lead to organ disorders such as brain, liver and kidney injury [3]. Countries with nutritional deficiencies, particularly in Asia and Africa, experience a higher incidence of preeclampsia, contributing to 10 percent of pregnancy-related deaths due to high blood pressure [4][5]. Although the mechanism of preeclampsia remains unknown, some evidence suggests a relationship with placentation and endothelial disorders [3].

Underlying conditions, including diabetes, hypertension and obesity are risk factors for preeclampsia [6]. Studies have indicated an association between preeclampsia and placental ischemia, leading to alterations in certain biomarkers and growth factors. For example, the plasma placental growth factor (PlGF) to sFlt-1 ratio is known to be altered in preeclampsia patients compared to healthy women [7]. Recent studies have presented conflicting findings regarding the relationship between serum levels of calcium (Ca) and magnesium (Mg) and preeclampsia. Winarno, Gatot N. Adhipurnawan et al, discovered that patients with preeclampsia exhibit significantly lower levels of Ca and Mg in their serum compared to healthy women [8].

Similarly, RKD Ephraime et al, reported similar results in both patients and the control group [9].

However, Golmohammad lou et al reported no significant difference between the two study groups [10]. To the best of our knowledge, no systematic review or meta-analysis has been conducted on this topic before. The aim of this study is to assess the levels of magnesium and calcium in preeclampsia patients and healthy pregnant women to discovering any relationship between alterations in trace elements and the risk of developing preeclampsia, and the severity of the disease.

## Materials and Methods

The current systematic review and meta-analysis were previously registered in PROSPERO under the code CRD42021251265.

Search Strategy

We conducted a comprehensive search of online databases, including PubMed/Medline, Scopus, Embase, Web of Sciences, and Cochrane library, to identify relevant articles from their inception up to July 2023. The search strategy utilized MeSH terms and keywords as follows: ("Pre-Eclampsia» OR "Pre-eclampsia" OR «Preeclampsia» OR "Pregnancy Toxemia" OR "Edema-Proteinuria-Hypertension Gestosis" OR "Edema Proteinuria" OR "Hypertension Gestosis" OR "Hypertension-Edema-Proteinuria Gestosis" OR "Hypertension Edema Proteinuria Gestosis" OR "Toxemia Of Pregnancy" OR "Toxemia of Pregnancies" OR "EPH Complex" OR"EPH Toxemias" OR "EPH Toxemia" OR "EPH Gestosis" OR "Preeclampsia Eclampsia 1" OR "Preeclampsia Eclampsia 1s" OR "Proteinuria-Edema-Hypertension Gestosis" OR "Proteinuria Edema Hypertension Gestosis" OR Toxemia OR "Pre-eclamptic Toxaemia" OR "Pre-eclamptic Toxemia" OR "Preclampsia Preeclamptic Toxaemia" OR "Preeclamptic Toxemia" OR "Pregnancy-Induced Hypertension" OR "Eclampsia» OR «Eclampsias» OR "HELLP Syndrome" OR "Syndrome HELLP" OR "Hypertension Pregnancy" OR "Hypertemsion Preeclampsia" OR "Gestational Hypertension" OR "Postpartum Hypertension-Preeclampsia" OR "Pregnancy-Related Hypertensive Disorders" OR "Toxemia in Pregnancy" OR "Hypertension in Pregnancy" OR "High Blood Pressure in Pregnancy" OR "Gestational Proteinuric Hypertension") AND ("Magnesium» OR «Mg2+» OR «Mg» OR "Magnesium Compounds" OR "Romag» OR «Magnesium Sulfate» OR «Magnesium Supplementation» OR «Magnesium Sulphate» OR «Mg Longoral» OR «Sulfamag» OR «Sulmetin» OR «Sulmetine» OR «Epsom Salt» OR «Epsom Salts» OR «Magnesium Sulphate in Dextrose 5" OR "Ca(2+) Mg(2+)-ATPase" OR "Mg2+-ATPase" OR "Mg2+ ATPase" OR "Mg2+-Dependent ATPase" OR "Mg2+ Dependent ATPase" OR "Calcium Magnesium ATPase" OR "Ca Mg-ATPase" OR "Ca Mg ATPase" OR "Ca2+-Mg2+ ATPase" OR "Ca2+ Mg2+ ATPase" OR "Calcium Magnesium Adenosine Triphosphatase" OR "Calcium Magnesium Adenosine Triphosphatase" OR "Magnesium Adenosine Triphosphatase" OR "Magnesium ATPase" OR "Magnesium Hydroxide" OR "Magnesium Hydrate" OR "Magnesium Deficiencies" OR "Magnesium Deficiency" OR "Magnesium Phosphate" OR "Magnesium Hydrogen Phosphate" OR "Magnesium Phosphate" OR «Magnesium Carbonate» OR «Magnesite» OR «Anhydrous Magnesium Carbonate» OR «Magnesium Carbonate Anhydrous» OR «Mg++» OR «Magnesium Ion» OR «Mg Ion» OR «Magnesium GTP» OR "Mg GTP» OR «Magnesium GTP" OR "Magnesia» OR "Magnesium Oxide" OR "Magnesium Chloride" OR «MgCl2» OR «Calcium» OR «Ca2+» OR «Ca» OR "Blood Coagulation Factor IV" OR "Coagulation Factor IV" OR «Calcium-40» OR "Calcium 40" OR "Factor IV" OR "Calcium Isotopes" OR "Calcium Radioisotopes" OR «Hypercalcemia» OR «Hypocalcemia» OR «40Ca» OR "Calcium Content" OR "Calcium Deposition" OR "Calcium Regulating Agents" OR "Calcium-Regulating Hormones and Agents" OR "Calcium Deposition"). To enhance the sensitivity of our search strategy, we also performed a manual search using the Google Scholar search engine and reviewed the reference lists of included studies and previous reviews.

Inclusion and Exclusion Criteria

We included all observational studies conducted in humans and published in English that met the following criteria: 1) included pregnant women of any gestational age; 2) compared serum calcium or magnesium levels between two groups (group 1: women presenting with pre-Eclampsia, group 2: women with a healthy pregnancy); and 3) reported at least one of the outcomes of interest related to calcium or magnesium.

We excluded studies such as previous review articles, case reports, case series, in vitro, in vivo studies, letters to the editor, commentaries, abstracts without full text, or studies with insufficient data.

Data Extraction

Two investigators (A.E & Z-K.M) independently extracted relevant data, including participant and outcome characteristics, using an Excel software spreadsheet for data abstraction. A third author (Sh.R) cross-checked the data to ensure accuracy. The extracted information included author names, study location, publication year, study method, sample size (in pre-eclamptic and healthy pregnant women), main participants’ characteristics, mean maternal age (in pre-eclamptic and healthy pregnant women), and key outcome data on mean and standard deviation (SD) of calcium and magnesium levels in both groups. Co-variables such as gestational age and body mass index (BMI) were also extracted. In some cases, articles were extracted multiple times due to the availability of subset data.

Risk of Bias (Quality) Assessment

Two investigators (A.E. and P.N.) independently assessed the quality of the included studies using the Newcastle-Ottawa Scale, which evaluates selection, comparability, and exposure/outcome aspects. Studies with a Newcastle-Ottawa Scale score of ≥5 for cross-sectional designs or a score of 7 or higher for case-control or cohort designs were considered to have good study quality (Suppl. Table-1s).

Statistical Analysis

The mean changes in serum calcium and magnesium levels in women with pre-eclampsia were estimated by calculating the weighted mean differences (WMDs) and 95% confidence intervals (CIs). Effect sizes were pooled using a random-effects model with the DerSimonian-Laird method for the meta-analysis. Heterogeneity among studies was assessed using Cochran’s Q and I-square tests. A Cochran’s Q test P-value of less than 0.1 and I-square value above 50% indicated significant heterogeneity. Sensitivity analysis was performed to evaluate the influence of individual studies on the final results. Publication bias among the included studies will be assessed using the Egger test and visual funnel plots. All statistical analyses were conducted using STATA software version 16.0 (Stata Corp., College Station, TX).

## Results

**Table:** Table1. Characteristics of Included Studies

**Authors** **(Publication year) **	**Country**	**Study type**	**Sample size** **(Case/control)**	**Body mass index ** **(case)**		**Body mass index ** **(control)**		**gestational age in case (weeks) **		**gestational age in control (weeks) **		**Maternal age in case (years) **		**Maternal age in control (years) **		**Quality** **Assessment**
				**mean**	**SD**	**mean**	**SD**	**mean**	**SD**	**mean**	**SD**	**mean**	**SD**	**mean**	**SD**	
Hamedanian et al. (2019) [88]	Iran	Case-control	60/60	27.9	4.9	24.3	3.9	32.4	4.4	34.5	4.8	31.5	5.3	29.0	5.3	**8**
Abbasalizadeh et al. (2019) [1]	Iran	Case-control	52/51	31.9	5.0	28.9	4.7					30.8	6.3	30.2	7.1	**7**
Ambad et al. (2020) [89]	India	Cross-sectional	100/100													**6**
Chaudhari et al. (2018) [18]	Nepal	Cross-sectional	37/37	29.3	5.4	24.1	3.7	36	2.9	31.2	4.3	26.7	5.4	25.9	4.9	**6**
Babacan et al. (2011) [90]	Turkey	Cohort	34/11					34.3	3.7	37.5	1.1	30.5	6.1	30	5.8	**8**
Dogan et al. (2021) [91]	Turkey	Case-control	42/46	24.6	3.2	24.3	3.1	36.5	2.4	38.8	0.9	31.7	5.9	31.5	5.3	**7**
Farzin et al. (2012) [27]	Iran	Case-control	60/60	27.1	3.2	26.8	2.2	35.5	1.1	35.3	1.2	27.4	3.9	26.7	3.7	**8**
Elmugabil et al. (2016) [24]	Sudan	Case-control	50/50	29	5	27	5.1	37.1	1	36.8	1	28.6	6.4	28.6	6.6	**7**
Hashemipour et al. (2017) [92]	Iran	Case-control	74/75													**8**
Golmohammad Lou et al. (2008) [10]	Iran	Case-control	52/52	21.6	50	21.4	51	35.2	0.8	36.7	1.1	25.7	1.2	22.7	1.5	**8**
Alghazali et al. (a) (2014) [93]	Iraq	Case-control	31/50	28.7	2.1	27.1	2.2					26.5	6.5	25.0	5.5	**7**
Alghazali et al. (b) (2014) [93]	Iraq	Case-control	19/50	30.3	3.9	27.1	2.2					28.5	6.7	25.0	5.5	**7**
M. E. Gunes et al. (2021) [28]	Turkey	Case-control	40/40	33.1	4.8	28.9	3.5	37.0	1.3	38.3	0.9	30.0	8.2	25.9	4.7	**7**
B. Adam et al. (2001) [11]	Turkey	Case-control	20/20					35	4	37	3.9	29	8	27	6.8	**8**
L. Poonia et al. (2021) [64]	India	Cross-sectional	100/100	24.5	3.7	22.3	2.9									**5**
Ahsan et al. (2013) [12]	Bangladesh	Cross-sectional	44/27					35.6	3.8	36.2	2.6	26.1	5.4	24.1	4.9	**6**
S. Akhtar et al. (2011) [13]	Bangladesh	cross sectional	60/30	25.8	2.4	23.3	2.1	32.3	3.5	31.5	3.9	25.1	5.7	25.2	4.9	**6**
Al-Rubaye et al. (a) (2009) [14]	Iraq	Cross-sectional	30/30													**5**
Al-Rubaye et al. (b) (2009) [14]	Iraq	Cross-sectional	30/30													**5**
R. Aziz et al. (2014) [15]	Pakistan	Case-control	16/16					32.3	4.8	32.9	5.8	24.7	17	25.6	58.9	**8**
Borekci et al. (2009) [16]	Turkey	Case-control	24/16					34.3	1.3	33.8	1.5	30.9	7.7	27.5	5.5	**6**
E. O. Darkwa et al. (2017) [20]	Ghana	cross sectional	30/30	32.0	7.5	30.5	5.5					30.9	5.5	29.9	2.6	**6**
B. Das et al. (2014) [21]	India	Case-control	40/40					31.2	4.1	33	4.4	25.9	3.4	26.5	2.8	**8**
A Dhungana et al. (2017) [23]	Nepal	Case-control	35/35													**5**
Talat J. Hassan et al. (1991) [29]	Pakistan	Case-control	50/100					36	3	36	3	22	3	22	3	**7**
E. S. Idogun et al. (2007) [30]	Nigeria	Cross-sectional	11/23									32	5.3	33	5.7	**6**
I. C. Ikechukwu et al. (2012) [31]	Nigeria	Cohort	59/150	29.4	4.6	27.6	3.7	35.5	2	39	1.6	27.3	3.2	26.7	3.6	**6**
S. Jain et al. (a) (2009) [34]	India	Case-control	25/50					34.9	3.5	33.6	7.8	23.0	3.8	23.9	3.4	**7**
S. Jain et al. (b) (2009) [34]	India	Case-control	25/50					35.1	3.6	33.6	7.8	22.9	3.8	23.9	3.4	**7**
B. Jamal et al. (2017) [35]	Pakistan	Cross sectional	40/40	25.3	0.4	23.5	0.3	35.3	0.4	36.8	0.3	25.8	0.7	25.5	0.8	**6**
D. V. Kanagal et al. (2014) [36]	India	Case-control	60/60	27.1	3.1	24.9	2.3	36.9	0.9	38.2	0.8	27.5	4.3	25.9	3.1	**8**
MK Kashyap et al. (2006) [37]	India	Case-control	100/100					34.3	3.7	38.3	1.2	25.9	3.7	25.4	2.4	**7**
O. Katz et al. (2012) [39]	Israel	Case-control	43/80					37.7	2.6	38.2	2.2	27.2	7.1	30.3	5.7	**7**
J. Kim et al. (2012) [42]	Korea	Case-control	29/30	24	5.8	21.3	3.3	34.1	3	39.1	1.1	32.1	4.6	31.9	3.1	**8**
K. Kisters et al. (2000) [43]	Germany	Case-control	16/18					35.2	2.1	33.8	2.4	28.8	6.7	27.8	5	**7**
K. Kisters et al. (1998) [45]	Germany	Case-control	20/25					34.9	2	33.6	2.2	27.5	6.3	28.7	5.1	**7**
K. Kisters et al. (1990) [49]	Germany	Case-control	27/22					35.1	2.2	33.7	2.3	27.3	6.1	29.6	4.7	**7**
M. Kosch et al. (2000) [50]	Germany	Case-control	16/18					35.2	2.1	33.8	2.4	28.8	6.7	27.8	5	**7**
S. Kumru et al. (2003) [51]	Turkey	Case-control	30/30									26.7	5.3	28	4.9	**7**
H. Lal et al. (1995) [53]	India	Case-control	25/25													**7**
J. Masse et al. (a) (1993) [55]	Canada	Cohort	109/1116	23.9	5.3	21.8	3.3	17.4	1.8	17.6	1.7	25.5	4.3	26.2	4.2	**6**
J. Masse et al. (b) (1993) [55]	Canada	Cohort	109/1136					29.1	1.2	29.3	1.5					**6**
S. Mittal et al. (2014) [56]	India	Case-control	100/100													**8**
K. Nahar et al. (2010) [57]	Bangladesh	cross sectional	20/60					35	20.1	38	18.4	25.4	6.2	25.3	4.3	**5**
C. E. M. Okoror et al. (2020) [59]	Nigeria	Case-control	27/54					33.4	3.9	33.5	3.6	32.1	6.5	32.2	6.1	**8**
E. B. Pedersen et al. (1984) [63]	Denmark	Case-control	15/18													**7**
C. Punthumapol et al. (a) (2008) [65]	Thailand	cross sectional	35/36	34.5	6.2	27.9	5.5	36.3	3.2	38.3	1.9	29.1	8.0	25.6	6.9	**6**
C. Punthumapol et al. (b) (2008) [65]	Thailand	cross sectional	33/36	27.3	8.9	27.9	5.5	36.2	3.6	38.3	1.9	25.6	7.0	25.6	6.9	**6**
D. G. D. Richards et al. (2013) [67]	South Africa	Case-control	96/96	28.6	8.4	28.4	8.4	20.9	6.5	21.8	6.8	24	4.3	24	4.4	**7**
S. R. Richards et al. (a) (1984) [68]	America	Case-control	20/16					35		38						**6**
S. R. Richards et al. (b) (1984) [68]	America	Case-control	11/16					34		38						**6**
M. Rostami et al. (2011) [69]	Iran	cross sectional	35/35													**4**
R. Sanders et al. (a) (1999) [70]	Netherlands	Case-control	15/6					32	4.3	13	1.8	28.7	5.2	31.2	6.2	**7**
R. Sanders et al. (b) (1999) [70]	Netherlands	Case-control	15/10					32	4.3	26.2	3	28.7	5.2	29.8	7	**7**
R. Sanders et al. (c) (1999) [70]	Netherlands	Case-control	15/18					32	4.3	33.9	2.5	28.7	5.2	31.1	4.7	**7**
C. A. Saputri et al. (a) (2020) [71]	Indonesia	Cross-sectional	30/30													**5**
C. A. Saputri et al. (b) (2020) [71]	Indonesia	Cross-sectional	12/30													**5**
P. P. Sende et al. (2019) [72]	Nigeria	Cross-sectional	90/90					36.4	2.5	36.2	2.3	28.7	5.2	28.3	5.1	**6**
C. Standley et al. (a) (1997) [73]	America	Case-control	9/22													**7**
C. Standley et al. (b) (1997) [73]	America	Case-control	9/22													**7**
C. Standley et al. (c) (1997) [73]	America	Case-control	9/22													**7**
K. Sukonpan et al. (2005) [75]	Thailand	Case-control	40/40	30.2	4.3	27.3	3.7	37.1	3	38.2	2	28.4	4.7	27	4.8	**8**
Z. Tavana et al. (2013) [76]	Iran	Cross-sectional	26/52					33.4	3.2	34.2	3.6	28.3	4.6	27.2	4.5	**6**
I. C. Udenze et al. (a) (2014) [78]	Nigeria	Case-control	50/50													**7**
I. C. Udenze et al. (b) (2014) [78]	Nigeria	Case-control	50/50													**7**
T. Fadhillah et al. (2021) [94]	Indonesia	Cross-sectional	40/40	26.7	5.3	22.7	3.2	32.6	5.1	37.5	1.2	30.5	6.8	33.1	4.9	**5**
M. I. Khattak et al. (2021) [40]	Pakistan	Case-control	40/40	31.1	2.0	28.7	2.1					27.8	4.2	28.2	4.9	**7**
D. D. Jain et al. (2021) [33]	India	Case-control	50/50									24.5	2.8	23.8	3.2	**8**
Kuye-Kuku TO et al. (2023) [52]	Nigeria	Case-control	60/60													**8**
I. K. P. Isong et al. (2022) [32]	Nigeria	Cross-sectional	30/30	33.4	6.6	30.3	3.6					28.7	5.3	30	5.4	**6**
M. Chauhan et al. (2021) [19]	India	Case-control	100/100									26.4	3.6	25.6	3.9	**8**
S. M. N. Uddin et al. (2022) [77]	Bangladesh	Case-control	74/118	26.7	2.5	25.6	1.7	31.0	6.1	25.9	5.9	27.5	8.4	24.6	5.5	**6**
R. Rani et al. (2022) [95]	India	Cross-sectional	37/17	29.3	5.4	24.1	3.7	36	2.9	31.2	4.3	26.7	5.4	25.9	4.9	**6**
W. R, Abdulhaleem et al. (2022) [2]	Iraq	Case-control	50/50					33.6	4.2	34.3	4.1	31.5	4.3	32.1	4.3	**8**
G. N. A. Winarno et al. (2021) [82]	Indonesia	Cross-sectional	138/108	30.6	5.2	28.1	5.3	36.3	4.1	36.4	3.8	30.7	8.2	30.3	6.7	**6**
S. Parvin et al. (a) (2021) [61]	Bangladesh	Case-control	40/40													**8**
S. Parvin et al. (b) (2021) [61]	Bangladesh	Case-control	40/40													**8**
R. D. Gebreyohannes et al. (2021) [5]	Ethiopia	Case-control	42/42	26.2	3.5	26.1	4.2	36.7	3.8	38.5	3.6	27	6	27	4	**6**
F. F. Khidri et al. (a) (2021) [41]	Pakistan	Cross-sectional	30/35					36.7	3.6	38.7	2.1	24.6	3.2	25.6	2.6	**6**
F. F. Khidri et al. (b) (2021) [41]	Pakistan	Cross-sectional	70/35					36.2	2.8	38.7	2.1	24.8	2.2	25.6	2.6	**6**
B. Rashid et al. (2015) [66]	Pakistan	Cross-sectional	100/100					35.5	1.8	36.5	1.3	26.1	2.6	25.7	1.8	**6**
S. Maksane et al. (2011) [54]	India	Case-control	20/20													**7**
f. Vahidroodsary et al. (2007) [81]	Iran	Case-control	50/50									24.1	5.2	27.8	6.4	**5**
J. Nnodim et al. (2017) [58]	Nigeria	Case-control	100/100	24.9	2.1	23.8	2.4	35.2	3.3	38.8	4.0	22.5	3.4	23.5	3.1	**8**
H.Vafaei et al. (a) (2015) [80]	Iran	Case-control	20/40													**8**
H.Vafaei et al. (b) (2015) [80]	Iran	Case-control	20/40													**8**
j. bringman et al. (2006) [17]	America	Case-control	10/10													**5**
M. Patwari et al. (2016) [62]	India	Case-control	50/100													**7**
O. A. Onyegbule et al. (2014) [60]	Nigeria	Cross-sectional	54/48	29.3	6.0	27.8	4.3					27	7.0	29	5.4	**6**
Ugwuja EI et al. (2016) [79]	Nigeria	Cross-sectional	40/40	20.3	3.9	27.2	5.4	21.4	3.2	21.5	3.7	29.5	3.7	27.6	4.2	**6**
R. Sujatha et al. (a) (2015) [74]	India	Case-control	40/50													**6**
R. Sujatha et al. (b) (2015) [74]	India	Case-control	10/50													**6**

**Figure-1:**
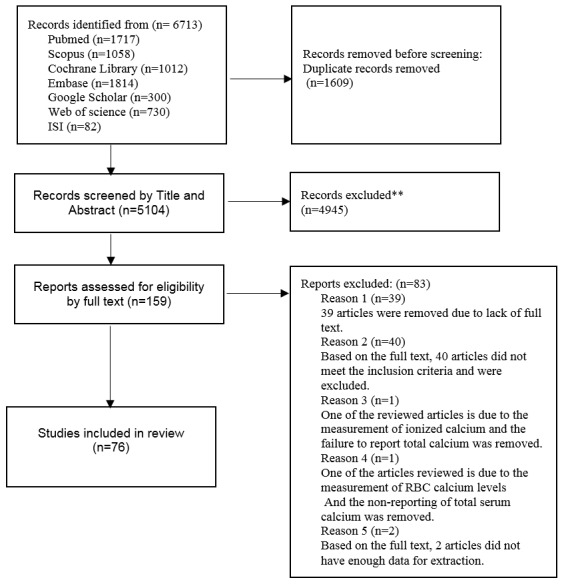
Figure 1. PRISMA flow diagram to study section

**Figure-2A:**
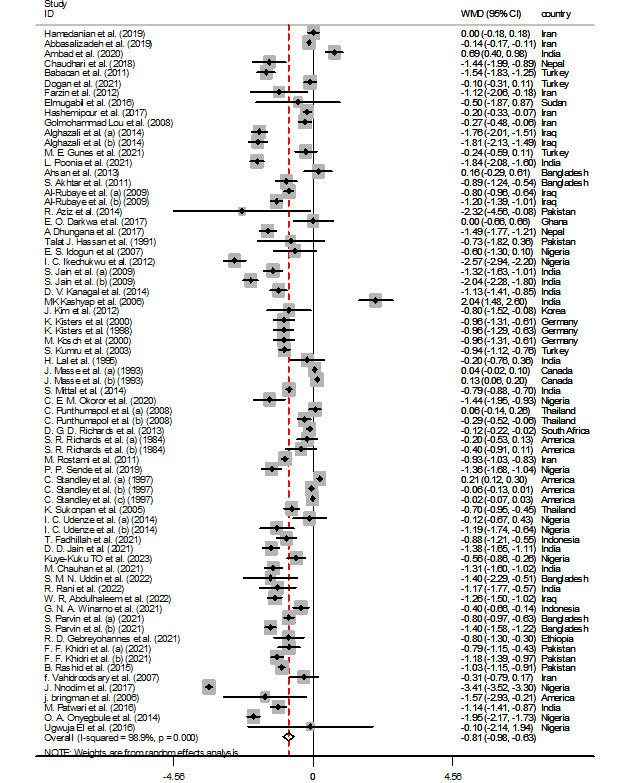
Figure 2A. The forest plot of meta-analysis of the association between calcium (A) and magnesium (B) levels with pre-eclampsia.

**Figure-2B:**
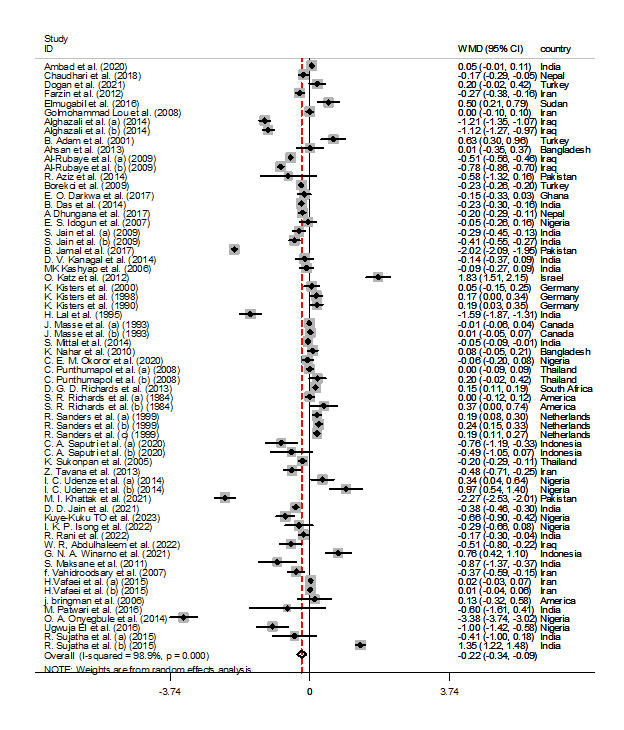
Figure 2B. The forest plot of meta-analysis of the association between calcium (A) and magnesium (B) levels with pre-eclampsia.

**Figure-3AB:**
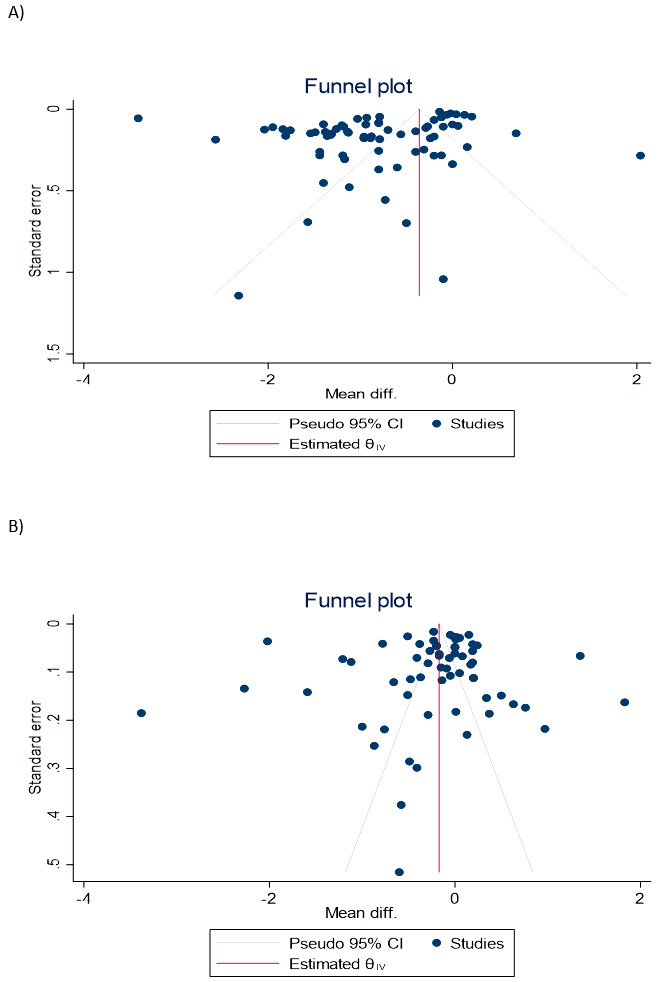
Figure 3AB. The funnel plots to visual assess of publication bias for calcium (A) and magnesium (B) levels across included studies.

Characteristics of Included Studies

Figure-1 illustrates the PRISMA flowchart depicting the step by step literature search and study selection process. After removing irrelevant and duplicate studies, we obtained the full-text papers of 159 articles out of 5104 for further evaluation based on the inclusion criteria. Among these, 83 articles did not adequately address the desired outcome and were therefore excluded. Ultimately, the meta-analysis was conducted based on 76 eligible articles (comprising 92 studies) [1][2][5][11][12][13][14][15][16][17][18][19][20][21][22][23][24][25][26][27][28][29][30][31][32][33][34][35][36][37][38][39][40][41][42][43][44][45][46][47][48][49][50][51][52][53][54][55][56][57][58][59][60][61][62][63][64][65][66][67][68][69][70][71][72][73][74][75][76][77][78][79][80][81][82]. Of these, 61studies were designed as case-control studies, 27 utilized a cross-sectional design, and 4 employed a cohort design. These studies included data from 10,482 pregnant women, with 3,991 in the pre-eclampsia group and 6,491 in the healthy pregnant women group. The included articles were published between 1984 and 2023. The key characteristics of these studies are summarized in Table-1.

Meta-analysis Outcomes

Based on the inclusion of 71 and 64 studies, respectively, the meta-analysis results, using a random-effects model, for the association between calcium and magnesium levels with pre-eclampsia are depicted in Figure-2A-B. The pooled analysis demonstrates a significant decrease in the levels of calcium (WMD=-0.807 mg/dL, 95% CI: -0.983, -0.632, P<0.01) and magnesium (WMD=-0.215, 95% CI: -0.338, -0.092, P<0.01) in women with pre-eclampsia compared to controls. Considering the observed heterogeneity among the included studies, a sensitivity analysis was conducted. However, excluding individual studies one by one did not result in any significant changes in the overall pooled WMD for calcium and magnesium levels.

Publication Bias

The funnel plots displaying calcium and magnesium levels are shown in Figure-3A-B. Statistical confirmation using Egger’s tests revealed no evidence of publication bias, as indicated by P-values of 0.63 and 0.25 for calcium and magnesium levels, respectively.

## Discussion

To the best of our knowledge, this is the first systematic review and meta-analysis to assess the relationship between serum calcium (Ca) and magnesium (Mg) levels and preeclampsia. The results of our study demonstrated that patients with preeclampsia had significantly lower levels of calcium (WMD=-0.807 mg/dL, 95% CI: -0.983, -0.632, P<0.01) and magnesium (WMD=-0.215, 95% CI: -0.338, -0.092, P<0.01) compared to the healthy control group. Recent studies have also reported alterations in some trace elements in preeclampsia. Our findings support the concept that serum calcium and magnesium levels are lower in preeclampsia compared to the healthy control group. Ephraim et al conducted a study involving 380 pregnant women and reported high blood pressure and lower serum levels of calcium and magnesium in preeclampsia patients [25].

Similarly, El-Maghraby et al recently revealed that both magnesium and calcium levels were decreased in preeclampsia patients [83]. Various theories have been proposed to explain the relationship between trace elements, particularly calcium and magnesium, and preeclampsia. The first theory is that some trace elements such as Ca and Mg can help alleviate oxidative stress by scavenging free radicals. Endothelial damage by oxidative stress is a key factor in the occurrence of preeclampsia. According to this, Ca and Mg have inevitable role in prevention of preeclampsia [84]. The second theory is that lower intake of calcium and magnesium is linked to increased blood pressure and the risk of preeclampsia due to the stimulation of hormonal system. The balance between calcium and magnesium serum levels is crucial for blood pressure control [5][85].

The third theory is that intracellular calcium concentration is increased in preeclampsia due to enhancement of absorbance by cells and the level of serum calcium is decreased, disturbance in the balance of intracellular and serum level of calcium lead to vasoconstriction and hypertension during pregnancy [43]. Therefore, it is important to maintain a balance in both serum and intracellular levels of Ca and Mg.

In conclusion, the prescription of calcium and magnesium supplements or multivitamins is recommended during pregnancy, especially for women at high risk of preeclampsia [86]. According to previous reports, the mean serum magnesium and total calcium levels in preeclampsia patients were 0.70±0.15 and 2.13±0.30 mmol/L, respectively, while in healthy pregnancies they were 0.76±0.14 and 2.13±0.35 mmol/L, respectively [87]. Due to endothelial dysfunction in preeclampsia, it is important to consider the role of interleukins and other inflammatory cytokines, which can be explored in future studies.

## Conclusion

In conclusion, our study, along with recent evidence, highlights the association between altered blood pressure and decreased levels of magnesium and calcium in preeclampsia. We found that the mean serum levels of magnesium and calcium were lower in patients with preeclampsia compared to the healthy control group. However, further studies are needed to investigate the levels of all trace elements in preeclampsia.

## Acknowledgment

This article received approval from the Ethics Committee of Fasa Medical University with codeIR.FUMS.REC.1400.145 and was supported by The Deputy of Research and Technology of Fasa University of Medical Sciences, Fasa, Iran, with grant number 400164.

## Conflict of Interest

The authors declare no conflict of interest.
